# Expectancy-value theories applied in Korean physical activity contexts: a meta-analysis

**DOI:** 10.3389/fpsyg.2025.1678503

**Published:** 2025-09-16

**Authors:** Jihyun Song, Wonseok Choi

**Affiliations:** Department of Kinesiology, Keimyung University, Daegu, Republic of Korea

**Keywords:** motivation, expectancy beliefs, task values, physical activity, meta-analysis

## Abstract

**Introduction:**

Recognizing expectancy-value theory as an influential framework for explaining the mechanism of motivation, relevant research has increased in physical activity contexts. This theory assumes that individuals’ motivation is situated and context-specific, shaped by the culture in which they live. Guided by expectancy-value theory, the purpose of this study was to synthesize the determinants and outcomes of motivation in Korean physical activity contexts through a meta-analytic approach.

**Methods:**

Following the PRISMA statement, this study performed a comprehensive literature search in five electronic databases by May 21, 2025. After extracting and collecting data from the included studies, a meta-analysis was carried out in the R software with the heterogeneity test, publication bias assessment, and subgroup analysis.

**Results:**

The findings demonstrated moderate-to-strong effects of the relationships between expectancy-value theory-based motivation and its determinants and outcomes, suggesting that individuals’ motivation was shaped by different internal and external factors, such as motivational constructs, social-contextual influences, and task difficulty, and contributed to affective experiences and behavioral engagement. Self-perceptions were likely to serve as a determinant of expectancy beliefs and an outcome of task values.

**Conclusion:**

These findings suggest both cross-cultural patterns and distinct mechanisms of expectancy-value theory-based motivation in physical activity contexts.

## 1 Introduction

Motivation in an achievement context is critical for engaging and sustaining goal-directed behaviors (Schunk and DiBenedetto, 2020). It is acknowledged that, motivation helps individuals facilitate physical activity (PA) and physical performance, which contributes to mental and physical health across different PA contexts such as school-based physical education (PE), college PE courses, and recreational PA (Penedo and Dahn, 2005; Shang et al., 2023). Among different motivation theories, expectancy-value theory (EVT) has provided a comprehensive understanding of motivational processes by capturing achievement-related effort, choices, and performances (Eccles and Wigfield, 2024). Developed by Eccles-Parsons et al. (1983), EVT consists of expectancy beliefs and task values. Expectancy beliefs refer to individuals’ beliefs about performing an upcoming task or activity well. These beliefs concern the question, “Do I think I can do the task?” reflecting one’s perceived competence. Task values represent internal and external incentives for engaging in the task and provide an answer to the question, “Why do I want to do this task?” Task values consist of four separate components: attainment value, intrinsic value, utility value, and cost (Eccles and Wigfield, 2024). Attainment value is the perceived importance of doing well on a provided task by incorporating one’s identity of ability. Individuals consider a task important when it is consistent with their own sense of identity. Intrinsic value is the anticipated enjoyment individuals would experience from doing a task. Individuals who attach high intrinsic value to an activity often become deeply engaged in and persist in it. Utility value is the perceived usefulness of doing a task to achieve one’s current or future goals. As this value is promoted when individuals perceive a task as aligned with their goals, and it is related to extrinsic motivation for performing the task. While task values facilitate individuals’ behavior, cost is the perceived negative component that devalues and limits participation in a task. Cost is identified as effort cost (the amount of effort required to perform a task), opportunity cost (the extent to which valuable alternatives must be given up to engage in a task), and emotional cost (the negative emotional states, such as anxiety, expected to arise while doing a task).

A merit of EVT is its ability to explain the complex interplay between EVT-based motivation (EVTM) and its determinants and outcomes. Eccles and Wigfield (2024) provided such determinants at individual and social-contextual levels. At an individual level, they include aptitudes, achievement-related experiences, previously formed affective memories, self-perceptions of one’s ability, and perceived task demands. At a social-context level, they encompass cultural milieu (e.g., gender role, stereotypes) and others’ (e.g., family, teachers, instructors, peers) beliefs and behavior. These determinations are considered to influence individuals’ expectancy beliefs and task values, leading to achievement-related choice and performance (Eccles and Wigfield, 2024). Consistent with this theoretical conceptualization, international research in PA contexts, mostly within PE, has clarified determinants that influence individuals’ expectancy beliefs and task values, such as self-perceptions (Beak et al., 2020; Kang et al., 2017; Kim and Yoon, 2023), influences of others (e.g., parents, teachers, peers) (Kim, 2010; Williams and Weiss, 2018), and PA context climate/social support (Gu et al., 2014; Jung, 2019; Kim, 2016; Zhang et al., 2012). The literature has also explored various outcomes, including, but not limited to, PA participation (Ding et al., 2013; Gu et al., 2012; Kim, 2010), intention to be physically active (Jung, 2019; Vernadakis et al., 2014), physical function and skills (Ding et al., 2013; Gu et al., 2012), learning achievement (Ding et al., 2013; Park, 2012), and persistence (Yang, 2019).

With the growing body of empirical research on EVTM applied to PA contexts, Shang et al. (2023) in a meta-analysis systematically synthesized previous studies, especially in elementary and secondary school PE in Western culture (e.g., Europe, U.S.). The study revealed different determinants of EVTM, including social support, teachers’ and peers’ EVTM, and class climate; and outcomes such as learning behaviors, situational interest, PA participation, and physical function and skills. These findings contributed to clarifying the mechanism of EVTM in PA contexts by identifying consistent patterns of its determinants and outcomes. Despite its contributions, the current understanding of EVTM applied to PA contexts remains incomplete because most of the studies included in the meta-analysis were conducted in Western culture (Shang et al., 2023). A question remains about whether its findings are applicable in non-Western cultural environments, such as South Korea.

According to Eccles and Wigfield (2024), at its inception, EVTM was developed with a strong emphasis on cultural influences. They argued that motivation is not shaped in a vacuum but is situated and context-specific within the very culture individuals live in. Supporting this perspective, Jang et al. (2023) compared college students’ motivation in the U.S. and South Korea and substantiated that U.S. students had higher perceived competence than Korean students. Similarly, Ahn et al. (2017) revealed that U.S. middle school students possessed higher perceived competence in mathematics than Korean students, with higher anxiety. They reasoned that these differences may reflect cultural distinctions. For example, U.S. students tend to overestimate their abilities, whereas Korean students often underestimate their abilities because of the cultural characteristics that emphasize humility. These findings imply that EVTM in Korean PA contexts may vary in its function in terms of determinants and outcomes, suggesting the need to synthesize the existing body of research in order to clarify how EVTM operates in this cultural environment.

Therefore, the purpose of this study was to synthesize determinants and outcomes of EVTM in Korean PA contexts through a meta-analytic approach. To fulfill this purpose, the study analyzed the relationship between EVTM and its determinants and outcomes. The findings of this study would contribute to understanding the mechanism of EVTM within the specific cultural environment and offer empirical evidence for future research when applying EVTM to PA contexts.

## 2 Methods

The review process was followed by the Preferred Reporting Items for Systematic Reviews and Meta-Analysis (PRISMA) guidelines (Page et al., 2021). We first established clear inclusion and exclusion criteria to identify previous studies. We then conducted a literature search using multiple electronic databases. Data were extracted and collected from the included studies, and the methodological quality was assessed. Last, we performed statistical analyses to synthesize the determinants and outcomes of EVTM applied to PA contexts.

### 2.1 Inclusion and exclusion criteria

Inclusion criteria for the literature search were as follows: (a) participants involved in a Korean PA context, (b) measured EVTM components, including expectancy beliefs, attainment value, intrinsic value, utility value, task values, and/or cost, (c) measured determinants and/or outcomes of EVTM, (d) reported at least one effect size (e.g., *r, β*) representing the association between EVTM and its determinants and/or outcomes, (e) statistics needed for analysis (e.g., *r*, *β*, sample size), and (f) peer-reviewed studies published in English and Korean. Studies that did not meet the inclusion criteria were excluded, including those with motivation constructs not relevant to EVT, qualitative studies, and those lacking statistics for analysis. In addition, non-peer-reviewed studies, such as unpublished articles, theses/dissertations, conference abstracts, and grey literature, were also excluded.

### 2.2 Literature search strategy

Following the inclusion and exclusion criteria, a structured electronic literature search was conducted in five electronic databases: Korea Citation Index, DBpia, Korean Studies Information Service System, Scholar, and eArticle. Search strings to identify eligible studies were “expectancy-value OR expectancy beliefs OR task value” AND “physical activity OR physical education OR sport” in English and Korean. Since this study was the first meta-analysis on EVTM applied to Korean PA contexts, no date restrictions were imposed. All peer-reviewed studies meeting the inclusion criteria and published by May 21, 2025 were included in the search process.

### 2.3 Data extraction and collection

To extract and collect data, we independently reviewed the eligible studies, following the established inclusion and exclusion criteria. We first reviewed 15 articles and developed an initial coding sheet. We then met to discuss each coding category of the coding sheet, resolving discrepancies. For example, we debated self-efficacy, often considered part of expectancy beliefs, and reviewed the relevant literature. In a subsequent meeting, we determined self-efficacy as a different motivational construct. This decision was based on the extensive literature review of EVTM and self-efficacy (e.g., Bandura, 1986), as well as conceptual distinctions (e.g., Zhu and Chen, 2010). We often communicated online when an emerging code was detected. Table 1 presents a summary of the coding sheet, including 54 codes classified into 11 code categories. In some cases where variables were theoretically and conceptually reversed (e.g., fixed belief vs. incremental belief), the effect sizes were adjusted to make the variables consistently integrated into the code categories (Borenstein et al., 2009). During this data extraction process, statistics (e.g., *r, β*) and relevant characteristics of the studies were also collected, including sample size, PA contexts, gender, and primary analysis. As seen in Table 1, PA contexts were classified into five types as follows: elementary school PE, secondary school PE, college PE, recreational center PA, and structured training/exercise. While most classifications followed the descriptions provided in the original studies, the structured training/exercise context was synthesized by the authors based on the shared characteristics of the contexts (e.g., skill development). The extracted data were then assigned to either the “determinant–EVTM pairs” spreadsheet or the “EVTM–outcome pairs” spreadsheet to conduct meta-analyses. The assignment adhered to the association descriptions from the original studies; if the function of EVTM was not clearly defined in the studies, decisions were made based on conceptual reasoning and empirical evidence.

**Table 1:** A summary of the coding sheet.

Code categories	Specific codes
PA contexts	Elementary school PE, secondary school PE, college PE, recreational center PA, structured training/exercise
Gender	Female, male, mixed
Engagement behavior	Engagement behavior, exercise persistence, intention to (re)engagement, intention to task persistence, light physical activity, moderate physical activity, skill practice, task involvement, task persistence
EVTM components	Expectancy beliefs, attainment value, intrinsic value, utility value, task values, cost
Motivational constructs	Autonomous motivation, intrinsic motivation, mastery goal, performance goal, perceived competence, self-efficacy, types of regulation (i.g., external, introjected, and identified regulations)
Affective experiences	Class satisfaction, interest in class, positive emotion, negative emotion
Self-perceptions	Fixed beliefs, incremental beliefs, mindset, perceived identity, self-perception
Social-contextual factor	Autonomy-supportive teaching, caring climate, class climate, controlled teaching, parental influence, peer influence, relatedness-supportive teaching
Learning achievement	Final grade
Task difficulty	Perceived task difficulty
Primary analysis	Latent class analysis, multi-group structured equation modeling, structured equation modeling, regression analysis

### 2.4 Quality assessment of the included studies

Quality of the included studies was assessed using the Quality Assessment Tool for Observational Cohort and Cross-Sectional Studies (National Institutes of Health [NIH], 2020). Of the 14 items in the tool, Items 3, 6, 7, 10, 12, and 13 were omitted because they were designed to assess observational cohort studies, which were less relevant to the purpose of this study. We assessed the quality of the included studies independently and coded each item as “Yes” if the criterion was met and “No” if it was not met. We coded “Partial” if the criterion was partially met. We had research meetings until all item ratings were reached through consensus. As an example of Item 11, which was designed to assess the presence of a clear definition, validity, reliability, and consistent implementation of outcome measures, we decided to rate it as “Partial” in some studies (e.g., Kim, 2010) that provided such information for only part of the outcome variables. Based on the aggregate number of “Yes” and “Partial” ratings, we rated the quality of the studies with the total scores of 6–8, 3–5, and 0–2 as “good,” “fair,” and “poor,” respectively.

### 2.5 Data analysis

Two commonly reported measures, correlation coefficients (*r*s) and standardized regression analysis coefficients (*β*s), were used to calculate effect sizes in the meta-analysis. *β*s were converted to *r*s to ensure consistency and comparability of effect sizes across studies. Following Peterson and Brown’s (2005) recommendation, *β*s ranging from −0.50 to 0.50 were computed, applying *λ* = 1 if *β* ≥ 0 and *λ* = 0 if *β* < 0. As *r*s are strongly influenced by variance, they were converted to Fisher’s *z* scores for the meta-analysis (Borenstein et al., 2009). Then, the *z*-scores were transformed back to *r*s when reporting the meta-analysis results. The transformed *r*s were interpreted as weak (>0.1), moderate (>0.3), and strong (>0.5) (Cohen, 1988).

The meta-analysis was conducted using the robumeta package in the R software (version 4.5.0). A random-effects model was applied with the assumption that the true associations between EVTM and its determinants and outcomes would differ study by study because of differences in study characteristics and populations (Borenstein et al., 2009). Acknowledging that effect sizes included in a study would be non-independent, this study used a robust variance estimator to yield more accurate estimates and avoid sample size inflation (Fisher and Tipton, 2015). In addition, at least two *r*s derived from more than one study were included in the meta-analysis to ensure statistical reliability. Heterogeneity was examined through *Q* and *I^2^* statistics. Significant *Q* values indicate potential heterogeneity, and *I^2^* values of <25, 25–50%, and >75% are interpreted as low, substantial, and considerable levels of heterogeneity, respectively (Higgins et al., 2003).

Assuming that specific types of PA contexts identified in the data extraction and collection processes might moderate the associations between EVTM and its determinants and outcomes, this study further performed subgroup analysis (Borenstein et al., 2009). The moderating effect of gender was not included in the analysis because of the insufficient sample sizes, raising concerns about the reliability and generalizability of moderator estimates. Publication bias was tested using funnel plots and Egger’s regression test. Sensitivity analysis was carried out through a leave-one-out analysis method, which determines whether one single study has a substantial influence on the overall effect.

## 3 Results

### 3.1 Study selection process

**Figure 1:**
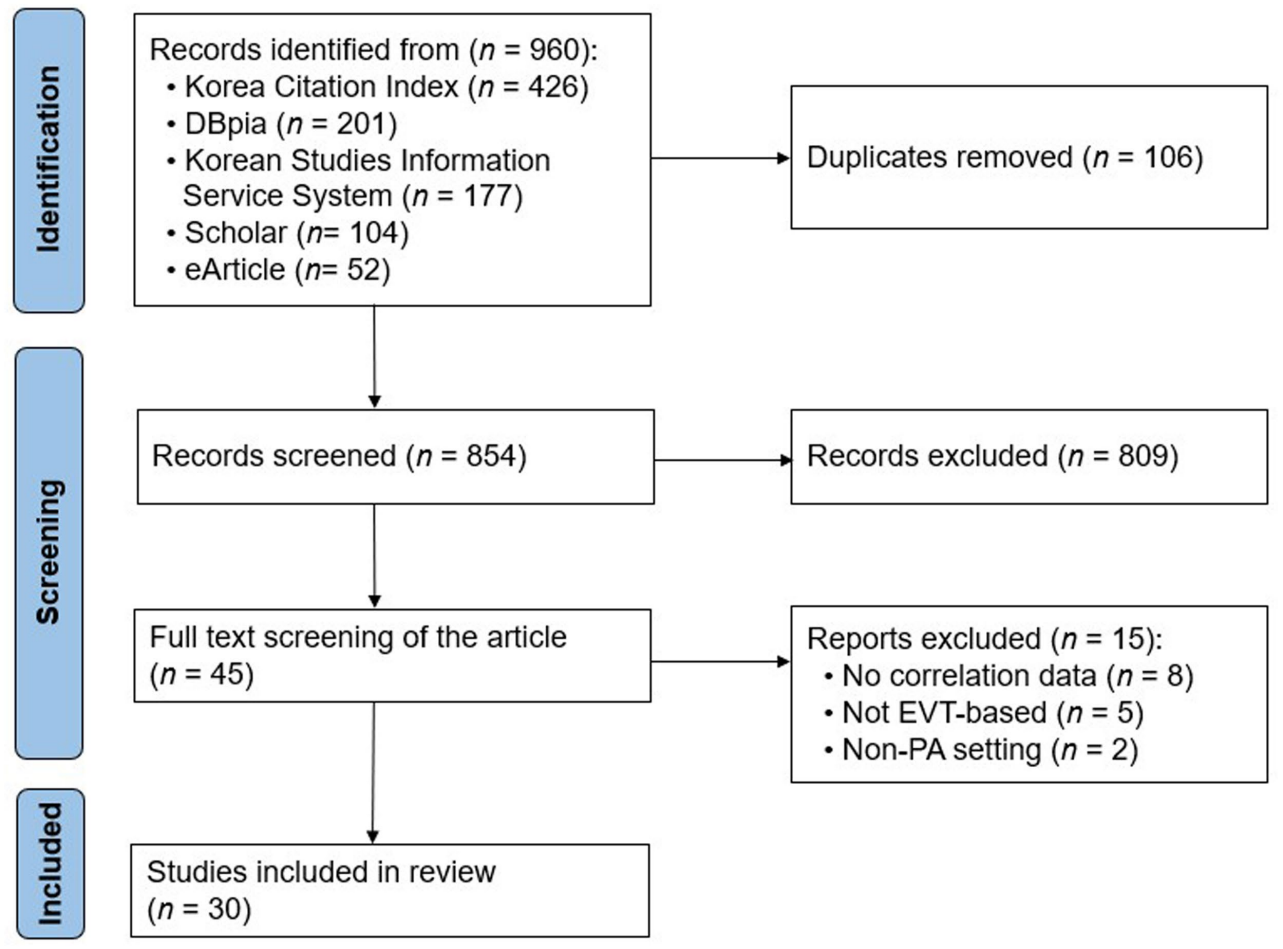
Flow diagram of literature search process.

Figure 1 exhibits the study selection process. In the initial database search, 960 articles were identified. After duplicates were removed, 854 articles were screened, of which 809 were excluded at the title and abstract level. The process resulted in 45 articles in the review pool. Then, the full texts were reviewed according to the inclusion criteria. In this reviewing process, 15 articles were excluded for the following reasons: no correlation data (*n* = 8), no EVTM (*n* = 5), and non-PA setting (*n* = 2). This reviewing process, in turn, yielded 30 articles for the meta-analysis.

### 3.2 Basic characteristics of the included studies

The included studies were reviewed and summarized to present their characteristics. They were all conducted since 2010, with 29 cross-sectional studies and one longitudinal study. PA contexts were identified as elementary PE (*n* = 5), secondary PE (*n* = 13), college PE (*n* = 6), structured training/exercise (*n* = 5), and recreational center PA (*n* = 1). The total sample size across the studies was 13,954 (*M* = 465.93, *SD* = 193.63), ranging from 155 to 901. Most studies (*n* = 27) included mixed-gender participants, while two involved only males (Choi, 2013) and females (Yang, 2019), respectively. One study (Park and Kim, 2012) included both genders but reported correlation coefficients separately. A majority of studies (*n* = 20) adopted structural equation modeling approaches with additional analysis, such as multi-group structural model analysis and latent class analysis, and the remaining ten (*n* = 10) used regression analysis. The Supplementary Table 1 describes the basic characteristics of the 30 studies, categorized by studies on the determinant–EVTM pairs and the EVTM–outcome pairs. In the 15 studies, EVTM served as both a determinant and an outcome, leading the basic characteristics of the studies to appear in both categories. Motivational constructs not based on EVT were the most commonly measured indicators in the relationship between determinants and EVTM (*n* = 8), while engagement behavior was the most frequently measured in the relationship between EVTM and outcome variables (*n* = 22).

### 3.3 Quality assessment of the included studies

Table 2 exhibits the overall quality assessment of the included studies. Most studies shared an obviously stated research question (Item 1); a clearly specified and defined study population (Item 2); study participants selected from similar populations with prespecified and uniformly applied inclusion/exclusion criteria (Item 4); different levels of independent variables (Item 8); a clearly defined independent variable and its validity, reliability, and consistent measurement (Item 9); and a clearly defined dependent variable and its validity and reliability, and consistent measurement (Item 11). However, many did not provide sample size justification, power description, or effect size estimates (Item 5), nor did they describe potential confounding variables measured and adjusted statistically (Item 14). All studies received 7–8 scores, suggesting good quality. Therefore, none of the studies were excluded in the quality assessment process, and they were all retained for the meta-analysis.

**Table 2:** Quality of the included studies.

Year	Authors	1	2	4	5	8	9	11	14	Rating
2024	Son and Yang, 2024	Y	Y	Y	N	Y	Y	Y	N	Good
2024	Yun, 2024	Y	Y	Y	N	Y	Y	Y	N	Good
2023	Kim and Yoon, 2023	Y	Y	Y	N	Y	Y	Y	P	Good
2023	Lee, 2023	Y	Y	Y	N	Y	Y	Y	N	Good
2023	Seo, 2023	Y	Y	Y	N	Y	Y	Y	N	Good
2024	Son and Yang, 2024	Y	Y	Y	N	Y	Y	Y	N	Good
2022	Gwak et al. 2022	Y	Y	Y	N	Y	Y	Y	Y	Good
2020	Beak et al., 2020	N	Y	Y	Y	N	Y	Y	Y	Good
2020	Jung and Lee, 2020	Y	Y	Y	N	Y	Y	Y	N	Good
2019	Jung, 2019	Y	Y	Y	N	Y	Y	Y	N	Good
2019	Lee, 2019	Y	Y	Y	N	Y	Y	Y	N	Good
2019	Song, 2019	Y	Y	Y	N	Y	Y	Y	N	Good
2019	Yang, 2019	Y	Y	Y	N	Y	Y	Y	N	Good
2018	Choi et al. 2018	Y	Y	Y	N	Y	Y	Y	N	Good
2017	Kang, 2017	Y	Y	Y	N	Y	Y	Y	Y	Good
2017	Kang et al., 2017	Y	Y	Y	N	Y	Y	Y	N	Good
2016	Jung, 2016	Y	Y	Y	N	Y	Y	Y	N	Good
2016	Kim, 2016	Y	Y	Y	N	Y	Y	Y	N	Good
2015	Cho, 2015	Y	Y	Y	N	Y	Y	Y	N	Good
2015	Jung and Nam, 2015	Y	Y	Y	N	Y	Y	Y	N	Good
2014	Do and Yoo, 2014	Y	Y	Y	N	Y	Y	Y	N	Good
2013	Choi, 2013	Y	Y	Y	N	Y	Y	Y	Y	Good
2013	Song and Huh, 2013	Y	Y	Y	N	Y	Y	Y	P	Good
2012	Park and Kim, 2012	Y	Y	Y	N	Y	Y	Y	P	Good
2014	Park and Yoo, 2014	Y	Y	Y	N	N	Y	Y	P	Good
2012	Park, 2012	Y	Y	Y	N	Y	Y	Y	P	Good
2011a	Park and Lee, 2011a	Y	Y	Y	N	Y	Y	Y	P	Good
2011b	Park and Lee, 2011b	Y	Y	Y	N	Y	Y	Y	P	Good
2010	Kim, 2010	Y	Y	Y	N	Y	Y	P	N	Good
2011	Park, 2011	Y	Y	Y	N	Y	Y	Y	P	Good

Y, yes; N, no; P, partial.

### 3.4 Meta-analysis of determinants of EVTM

#### 3.4.1 Publication bias and sensitivity analysis

**Figure 2:**
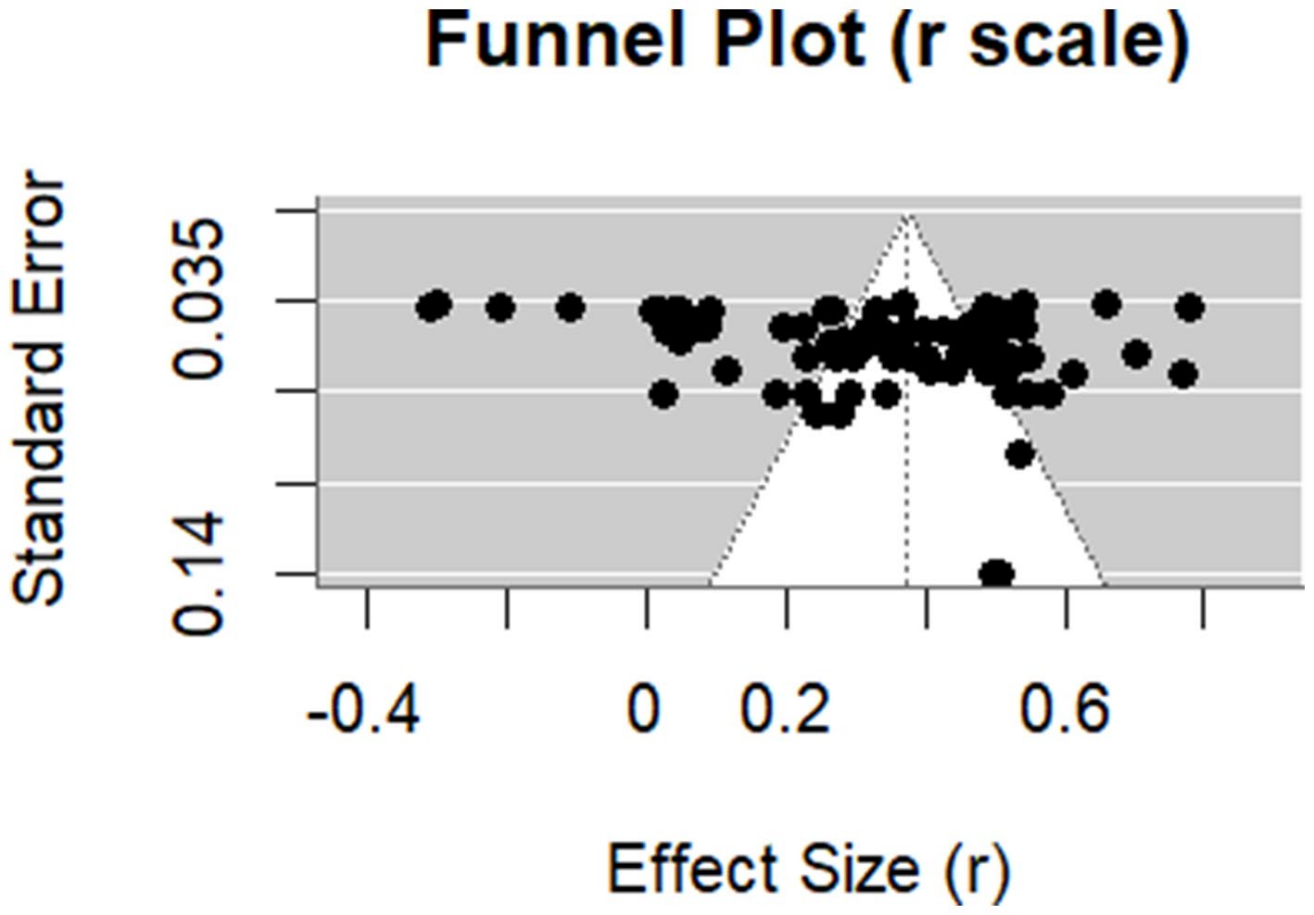
Funnel plot for determinants of EVTM.

Potential publication bias was assessed using a funnel plot and the Egger’s regression test. As shown in Figure 2, the funnel plot displays an asymmetric distribution supported by Egger’s regression test (*z* = 2.17, *p* = 0.03), implying potential publication bias. However, a leave-one-out analysis yielded stable mean effect sizes with no obvious outliers ( $\bar{r}s$ = 0.36–0.37). The result suggests that while publication bias may be present, no single study significantly impacted the overall result, and all included studies could be used for subsequent analyses.

#### 3.4.2 Overall effect size

Using a total of 80 *r*s obtained from 18 studies, the overall effect size was estimated as $\bar{r}$ = 0.36 (95% CI = [0.30, 0.42], *p* < 0.001), indicating a moderate effect of determinants on EVTM. The heterogeneity test results revealed statistically significant variation in effect sizes (*Q* = 2831.97, *p* < 0.001), but the portion of true variance was low (*I^2^* = 22.3%) (Higgins et al., 2003).

#### 3.4.3 Determinant–EVTM pairs

Table 3 presents the results of meta-analyses (*k* ≥ 2) for 14 determinants–EVTM pairs. Six pairs (i.g., motivational constructs–expectancy beliefs, motivational constructs–task values, self-perceptions–attainment value, self-perceptions–task values, social-contextual factors–expectancy beliefs, and social-contextual factors–utility task) demonstrated positively significant moderate-to-strong relationships ( $\bar{r}s$ = 0.38–0.68, 95% CIs = [0.02, 0.87]). The task difficulty–task values pair was also statistically significant, but the direction was negative. Seven pairs were not statistically significant, considering the 95% CIs crossing zero. Among the significant determinant–EVTM pairs, only the motivational constructs–expectancy beliefs pair demonstrated high heterogeneity (*Q_w_* = 194.05, *p* < 0.001, *I^2^* = 98.1%). The remaining pairs did not exhibit observable heterogeneity (*I^2^*s = 0%), and three of them showed statistically non-significant *Q_w_* statistics (*Q_ws_* = 0.55–5.07, *ps* > 0.05), suggesting a small amount of variance in effect sizes. However, it should be noted that the variance might be attributed to the small sample sizes, limiting the power to detect true variation (Higgins et al., 2003). In addition, central to EVT is the assumption that individuals’ motivation differs depending on the very situation or context individuals interact with, and thus, a subgroup analysis was conducted using the PA context type as a moderator.

**Table 3:** Determinant–EVTM pairs.

Determinant	EVTM	*k*	$\bar{r}$	±95% CI	*Q_w_*	*p*	*I^2^*
Motivational constructs	EB	4	0.68	[0.33, 0.87]	194.05	<0.001	98.1
	IntrinsicV	2	0.43	[−0.71, 0.95]	8.90	0.003	88.5
	TaskV	5	0.56	[0.42, 0.67]	36.01	<0.001	0
Self-perceptions	EB	6	0.34	[−0.04, 0.64]	61.18	<0.001	83.1
	AttainV	10	0.40	[0.21, 0.55]	173.28	<0.001	0.0
	IntrinsicV	9	0.31	[−0.38, 0.77]	161.81	<0.001	91.5
	UtilityV	8	0.34	[−0.17, 0.71]	163.34	<0.001	71.3
	TaskV	6	0.39	[0.15, 0.60]	24.50	<0.001	0
	Cost	2	0.32	[−0.70, 0.88]	33.09	<0.001	96.8
Social-contextual factors	EB	3	0.34	[0.02, 0.65]	0.55	0.459	0
	AttainV	4	0.29	[−0.85, 0.95]	45.62	<0.001	0
	IntrinsicV	4	0.31	[−0.96, 0.98]	47.37	<0.001	94.4
	UtilityV	4	0.53	[0.23, 0.74]	0.60	0.440	0
Task difficulty	TaskV	3	−0.27	[−0.49, −0.03]	5.07	0.080	0

EB, expectancy beliefs; attainV, attainment value; intrinsicV, intrinsic value; taskV, task values; utilityV, utility value.

#### 3.4.4 Subgroup analysis

Table 4 presents the results of the subgroup analysis. The *Q_b_* statistic was 133.63 (*p* < 0.001), indicating that the effect sizes varied across different types of contexts. All PA context types displayed statistically significant, moderate effects on the relationship between determinants and EVTM. The mean effect size was $\bar{r}$ = 0.27, 95% CI = [0.12, 0.41] in elementary school PE; $\bar{r}$ = 0.38, 95% CI = [0.20, 0.53] in secondary school PE; and $\bar{r}$ = 0.39, 95% CI = [0.31, 0.47] in the structured training/exercise setting. College PE and recreational center PA were excluded from this subgroup analysis due to the insufficient sample size.

**Table 4:** A summary of subgroup analysis results for the determinant–EVTM pairs.

Context	*k*	$\bar{r}$	±95% CI	*Q_w_*	*I^2^*	*p*	*Q_b_*
Elementary PE	23	0.27	[0.12, 0.41]	528.41	95.8	<0.001	133.63 (*p* < 0.001)
Secondary PE	24	0.38	[0.20, 0.53]	1966.22	98.8	<0.001	
Structured training/exercise	29	0.39	[0.31, 0.47]	188.18	85.1	<0.001	

Table 5 presents the mean effect sizes of the specific determinant–EVTM pairs within each context type as follows: the motivational constructs–expectancy beliefs ( $\bar{r}$ = 0.68, 95% CI = [0.33, 0.87]), motivational constructs–task values ( $\bar{r}$ = 0.56, 95% CI = [0.42, 0.67]), and task difficulty–task values pairs ( $\bar{r}$ = − 0.27, 95% CI = [−0.49, −0.03]) in secondary school PE; and the self-perceptions–expectancy beliefs ( $\bar{r}$ = 0.40, 95% CI = [0.03, 0.67]) and self-perceptions–utility value pairs ( $\bar{r}$ = 0.41, 95% CI = [0.12, 0.64]) in the structured training/exercise setting. None of the determinant–EVTM pairs in elementary school PE were statistically significant. In addition, most pairs exhibited substantial heterogeneity across the contexts (*Q_ws_* = 15.35–194.05, *ps* < 0.01, *I^2^s* = 73.9–98.5%), while the task difficulty–task values pair in secondary school PE reported no heterogeneity (*Q_w_* = 5.07, *p* > 0.05, *I^2^* = 0).

**Table 5:** Detailed subgroup analysis results for the determinant–EVTM pairs.

Context	Determinant	EVTM	*k*	$\bar{r}$	±95% CI	*Q_W_*	*p*	*I^2^*
Elementary PE	Self-percept	AttainV	6	0.37	[−0.32, 0.81]	148.26	<0.001	96.6
	Self-percept	IntrinsicV	5	0.22	[−0.79, 0.91]	79.36	<0.001	95.0
	S-C factors	AttainV	3	0.22	[0.14, 0.29]	31.08	<0.001	93.6
	S-C factors	IntrinsicV	3	0.16	[−0.44, 0.66]	19.50	<0.001	89.7
	S-C factors	UtilityV	2	0.38	[−0.97, 0.95]	41.53	<0.001	97.6
Secondary PE	Motiv	EB	4	0.68	[0.33, 0.87]	194.05	<0.001	98.5
	Motiv	TaskV	5	0.56	[0.42, 0.67]	36.01	<0.001	88.9
	Task difficulty	TaskV	3	−0.27	[−0.49, −0.03]	5.07	0.079	0.0
Structured training/exercise	Self-percept	EB	5	0.40	[0.03, 0.67]	21.72	<0.001	81.6
	Self-percept	UtilityV	3	0.41	[0.12, 0.64]	15.35	0.004	73.9
	Self-percept	TaskV	3	0.44	[−0.70, 0.95]	12.41	0.002	83.9

EB, expectancy beliefs; attainV, attainment value; intrinsicV, intrinsic value; motiv, motivational constructs; self-percept, self-perceptions; taskV, task values; utilityV, utility value.

### 3.5 Meta-analysis of outcomes of EVTM

#### 3.5.1 Publication bias and sensitivity analysis

**Figure 3:**
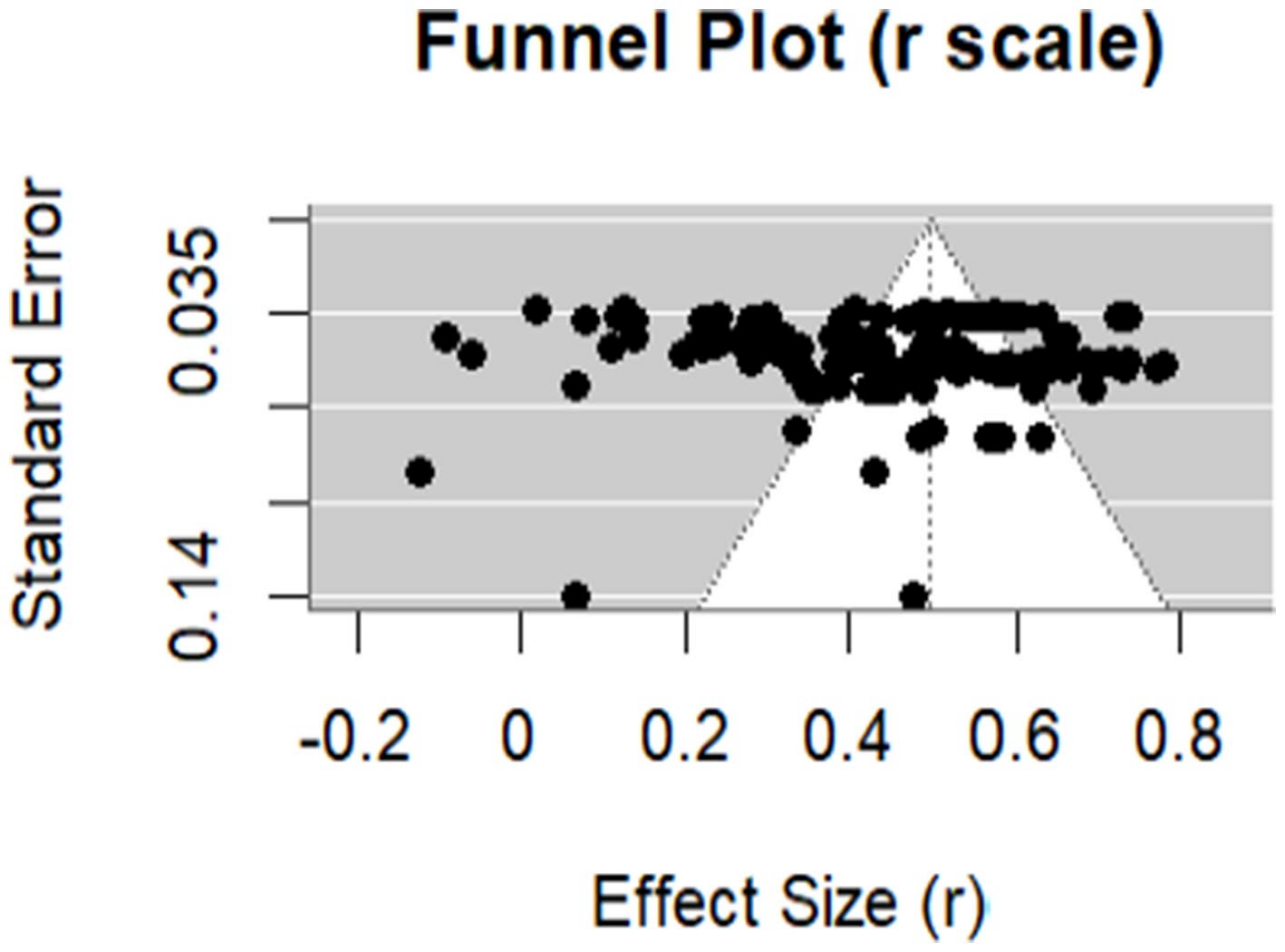
Funnel plot for outcomes of EVTM.

A funnel plot and the Egger’s regression test were used to assess publication bias. The funnel plot in Figure 3 and the Egger’s regression test result (*z* = −0.15, *p* = 0.88) exhibited minimal risk of publication bias in the included studies. A leave-one-out analysis yielded stable effect sizes without evident outliers (*r*s = 0.46–0.47), indicating that no single study would significantly influence the overall result. These findings allowed all included studies to be used in subsequent analyses.

#### 3.5.2 Overall effect size

A total of 149 *r*s derived from 27 studies were used to calculate the overall effect of the association between EVTM outcome variables. Substantial heterogeneity was found in the included studies (*Q* = 3694.37, *p* < 0.001, *I^2^* = 92.1%) (Higgins et al., 2003). The meta-analysis revealed the overall effect size of $\bar{r}$ = 0.46 (95% CI = [0.39, 0.53], *p* < 0.001), indicating a moderate-to-strong influence of EVTM on relevant outcomes in PA contexts.

#### 3.5.3 EVTM–outcome pairs

Table 6 displays the results of the meta-analyses (*k* ≥ 2) for 14 EVTM–outcome pairs. Four pairs (i.g., expectancy beliefs–motivational constructs, attainment value–affective experiences, task values–learning achievement, and cost–self-perceptions) were excluded due to the insufficient number of effect sizes. Nine pairs demonstrated statistically significant, moderate-to-strong effects ( $\bar{r}s\;$ = 0.41–0.60, 95% CIs = [0.19, 0.70]), suggesting that EVTM served as a determinant of outcomes, such as affective experiences, engagement behavior, and self-perceptions. Most of them showed at least substantial heterogeneity (*Q_ws_* = 79.66–739.73, *I^2^* = 53.5–96.7%). Two displayed a statistically significant *Q_w_* statistics (*Q_ws_* = 71.87–141.31, *ps* < 0.001) with *I^2^s* of 0% (Higgins et al., 2003), implying that the observed heterogeneity might reflect variation not accounted for in the analysis. Six pairs (i.g., expectancy beliefs–motivational constructs, utility–affective experiences, task values–motivational constructs, task values–self-perceptions, and cost–engagement behavior) did not yield statistically significant results with 95% CIs crossing zero.

**Table 6:** EVTM–outcome pairs.

EVTM	Outcome	*k*	$\bar{r}$	±95% CI	*Q_w_*	*p*	*I^2^*
EB	Affect	16	0.45	[0.19, 0.65]	739.73	<0.001	96.7
	Behavior	20	0.46	[0.40, 0.51]	130.98	<0.001	66.1
	Motiv	6	0.31	[−0.78, 0.69]	253.77	<0.001	99.4
	Self-percept	6	0.53	[0.31, 0.70]	141.31	<0.001	0
AttainV	Behavior	14	0.41	[0.29, 0.51]	247.24	<0.001	78
Intrinsic value	Affect	7	0.60	[0.59, 0.61]	71.87	<0.001	0
	Behavior	15	0.47	[0.37, 0.55]	179.71	<0.001	90.3
UtilityV	Affect	7	0.58	[−0.24, 0.92]	37.91	<0.001	85.7
	Behavior	13	0.47	[0.37, 0.56]	205.39	<0.001	53.5
TaskV	Affect	14	0.52	[0.36, 0.65]	430.12	<0.001	86.0
	Behavior	12	0.43	[0.31, 0.54]	79.66	<0.001	78.4
	Motiv	6	0.35	[−0.37, 0.81]	308.92	<0.001	0
	Self-percept	3	0.53	[−0.07, 0.85]	48.53	<0.001	95.8
Cost	Behavior	3	0.25	[−0.23, 0.63]	25.21	<0.001	91.8

Affect, affective experiences; attainV, attainment value; behavior, engagement behavior; intrinsicV, intrinsic value; motiv, motivational constructs; self-percept, self-perceptions; taskV, task values; utilityV, utility value.

#### 3.5.4 Subgroup analysis

As seen in Table 7, the *Q_b_* statistic was estimated as 318.57 (*p* < 0.001), suggesting that the effect sizes differed across types of PA contexts. The mean effect sizes were moderate to strong, ranging from $\bar{r}$ = 0.34 to 0.50 (95% CIs = [0.14, 0.61]). The strongest moderating effect of EVTM on outcome variables was found in secondary school PE ( $\bar{r}$ = 0.50, 95% CI = [0.37, 0.61]), followed closely by college PE ( $\bar{r}$ = 0.49, 95% CI = [0.39, 0.58]), elementary school PE ( $\bar{r}$ = 0.35, 95% CI = [0.14, 0.53]), and structured training/exercise settings ( $\bar{r}$ = 0.34, 95% CI = [0.15, 0.50]). Recreational center PA was excluded from the analysis because all effect sizes were obtained from a single study (i.g., Son and Yang, 2023), which did not ensure a robust pooled effect. All pairs across the contexts displayed high heterogeneity (*Q_ws_* = 108.98–2939.22, *ps* < 0.001, *I^2^s* = 79.6–97.5). These results imply the possibility that such variation in each context may be derived from specific pairs of EVTM components and outcomes, and accordingly, a subsequent subgroup analysis was conducted.

**Table 7:** A summary of subgroup analysis results for the EVTM–outcome pairs.

Context	*k*	$\bar{r}$	95% CI	*Q_w_*	*I^2^*	*p*	*Q_b_*
Elementary PE	18	0.35	[0.14, 0.53]	108.98	84.4	<0.001	318.57 (*p* < 0.001)
Secondary PE	75	0.50	[0.37, 0.61]	2939.22	97.5	<0.001	
College PE	31	0.49	[0.39, 0.52]	147.2	79.6	<0.001	
Structured training/exercise	21	0.34	[0.15, 0.50]	180.41	88.9	<0.001	

Table 8 exhibits moderate-to-strong mean effects for the EVTM–outcome pairs by type of PA context. Statistically significant pairs in each type were as follows: the attainment value–engagement behavior ( $\bar{r}$ = 0.29, 95% CI = [0.12, 0.45]) and intrinsic value–engagement behavior pairs ( $\bar{r}$ = 0.37, 95% CI = [0.17, 0.54]) in elementary school PE; the expectancy beliefs–affective experiences ( $\bar{r}$ = 0.45, 95% CI = [0.12, 0.69]), expectancy beliefs–engagement behavior ( $\bar{r}$ = 0.53, 95% CI = [0.40, 0.65]), and task values–affective experiences pairs ( $\bar{r}$ = 0.53, 95% CI = [0.34, 0.68]) in secondary school PE; the expectancy beliefs–engagement behavior pairs ( $\bar{r}$ = 0.45, 95% CI = [0.38, 0.51]) and utility value–engagement behavior pairs ( $\bar{r}$ = 0.51, 95% CI = [0.42, 0.58]) in college PE; and expectancy beliefs–behavioral outcome pair in structured training/exercise ( $\bar{r}$ = 0.35, 95% CI = [0.23, 0.45]). EVTM components were significantly associated with behavioral outcomes across the contexts, while they were associated with affective experiences in only secondary school PE. For heterogeneity, only two pairs (expectancy beliefs–engagement behavior and utility value–engagement behavior pairs) in college PE showed non-significant *Qw* statistics (*ps* > 0.05), while their *I^2^* values were relatively high (70.0 and 84.3%, respectively), implying relatively stable effects across studies.

**Table 8:** Detailed subgroup analysis results for the EVTM–outcome pairs.

Context	EVTM	Outcomes	*k*	$\bar{r}$	±95% CI	*Q_w_*	*p*	*I^2^*
Elementary PE	AttainV	Behavior	6	0.29	[0.12, 0.45]	18.69	<0.001	73.2
	IntrinsicV	Behavior	6	0.37	[0.17, 0.54]	39.25	<0.001	87.3
	UtilityV	Behavior	4	0.34	[−0.02, 0.61]	17.14	<0.001	82.5
Secondary PE	EB	Affect	14	0.45	[0.12, 0.69]	739.37	<0.001	98.2
	EB	Behavior	6	0.53	[0.40, 0.65]	81.36	<0.001	93.9
	EB	Motiv	6	0.31	[−0.06, 0.62]	253.77	<0.001	98
	EB	Self-percept	3	0.55	[−0.20, 0.89]	133.53	<0.001	98.5
	AttainV	Behavior	2	0.60	[−0.55, 0.96]	11.38	<0.001	91.2
	IntrinsicV	Behavior	2	0.63	[−0.91, 0.92]	36.07	<0.001	97.2
	UtilityV	Behavior	2	0.67	[−0.95, 0.96]	49.13	<0.001	98
	TaskV	Affect	12	0.53	[0.34, 0.68]	435.4	<0.001	97.5
	TaskV	Motiv	6	0.35	[−0.37, 0.81]	308.92	<0.001	35.8
College PE	EB	Behavior	3	0.45	[0.38, 0.51]	7.78	0.169	84.3
	EB	Self-percept	3	0.52	[−0.66, 0.96]	12.75	<0.001	0
	UtilityV	Behavior	3	0.51	[0.42, 0.58]	1.16	0.560	73
	TaskV	Behavior	5	0.45	[−0.46, 0.90]	7.41	0.025	70.3
Structured training/exercise	EB	Behavior	3	0.35	[0.23, 0.45]	13.45	0.009	96.5
	AttainV	Behavior	3	0.36	[−0.96, 0.99]	57.51	<0.001	84.4
	IntrinsicV	Behavior	3	0.41	[−0.30, 0.83]	12.8	<0.001	92.9
	UtilityV	Behavior	4	0.42	[−0.67, 0.94]	28.19	<0.001	87.8
	TaskV	Behavior	6	0.27	[−0.43, 0.77]	24.65	<0.001	73.2

Affect, affective experiences; attainV, attainment value; behavior, engagement behavior; intrinsicV, intrinsic value; motiv, motivational constructs; self-percept, self-perceptions; taskV, task values; utilityV, utility value.

## 4 Discussion

The purpose of this study was to synthesize determinants and outcomes of EVTM in Korean PA contexts through a meta-analytic approach. The findings of this study revealed that EVTM was shaped by multiple determinants, contributing to various outcomes. The type of PA context served as a moderator of the associations between EVTM and its determinants and outcomes. These findings paint a clear picture of a mechanism of EVTM in Korean PA contexts. Nevertheless, caution must be taken in interpreting these results because of substantial heterogeneity and the lack of sample size justification in the included studies.

### 4.1 Contribution of determinants to EVTM

The result of this meta-analysis demonstrated a moderate overall effect on the relationship between determinants and EVTM ( $\bar{r}$ = 0.36). The determinants included motivational constructs, self-perceptions, social-contextual factors, and task difficulty. Among these determinants, motivational constructs exhibited the strongest effects on both expectancy beliefs and task values, suggesting that individuals with higher autonomous motivation, self-efficacy, and achievement goals in PA contexts seem to facilitate their expectancy beliefs and/or task values. However, international research in PA contexts, specifically in Western culture, has often provided empirical evidence for the opposite direction of the relationship, which is the contributor of EVTM to motivational constructs. For example, Zhu and Chen (2015) revealed that students’ expectancy beliefs in secondary school PE predicted self-efficacy. Shang et al. (2023) in a meta-analysis also proved that EVTM contributed to situational interest and self-efficacy. With the evidence, the findings of this study imply that EVTM may be bidirectional or reciprocal in nature in relation to motivational constructs, rather than strictly unidirectional, reflecting a complex interplay of motivational constructs and EVTM.

Self-perceptions also served as determinants of attainment value and task values. In the included studies, some (e.g., Beak et al., 2020; Yun, 2024) measured attainment value, intrinsic value, and utility value, respectively, while others (e.g., Jung and Lee, 2020; Son and Yang, 2024) used the aggregated score of task values. The meta-analysis result revealed that both attainment value and task values were influenced by self-perceptions, but others, such as intrinsic value and utility value, were not. These findings imply that attainment value may be the EVTM component most strongly worked with self-perceptions. Attainment value often reflects how individuals perceive their own ability, which might lead them to consider a task something personally meaningful and important to who they are (Eccles and Wigfield, 2024).

Social-contextual factors were determinants of expectancy beliefs and utility value. These results suggest that the more individuals perceive the PA context they participate in as supportive and caring from others, such as teachers, parents, and peers, the more they are likely to believe they can success in a given task and to perceive the task as useful because it aligns with the current or future goals. In fact, the literature has underscored social-contextual factors as key to promoting individuals’ expectancy beliefs and task values (Eccles and Wigfield, 2024). International studies have provided empirical evidence for the impact of social-contextual factors, such as class climates and teacher, peer, and parent influences on expectancy beliefs and task values, primarily utility value (Shang et al., 2023; Williams and Weiss, 2018). With these findings, the results of this study suggest that the influence of social-contextual factors on EVTM is applicable to PA contexts in and beyond South Korea.

Perceived task difficulty negatively contributed to task values. It is interpreted that when performing a challenging task, individuals tend to decline the importance, interest, and usefulness of the task. Consistent with this interpretation, Li et al. (2007) revealed that the more students perceived tasks as difficult, the more they were likely to decrease EVTM in secondary school PE. According to Vygotsky (1978), tasks that are far beyond an individual’s current level of capability would hinder motivation, but tasks that are slightly more difficult but achievable with appropriate support would promote motivation. Thus, gradually increasing task difficulty through repeated practice may help individuals in PA contexts perceive the tasks as challenging yet attainable, promoting EVTM.

The subgroup analysis confirmed that elementary school PE, secondary school PE, and structured training/exercise served as a significant moderator in the association between determinants and EVTM. In particular, social-contextual factors impacted attainment value in elementary school PE; motivational constructs and task difficulty contributed to expectancy beliefs and task values in secondary school PE; and self-perceptions influenced expectancy beliefs and utility value in structured training/exercise settings. These findings suggest that the function of EVTM varies in specific types of PA contexts. Characteristics of PA contexts, such as content, goals of PA participation, instructional style, instructors’ and participants’ gender, and interactions with others, may influence the relationship between antecedents and EVTM within the context (Eccles and Wigfield, 2024). Recognizing these differences in each type of context is essential for developing theory-informed and context-sensitive strategies to support motivation in PA contexts.

### 4.2 Contribution of EVTM to outcomes

EVTM had a moderate-to-strong effect on outcomes ( $\bar{r}$ = 0.46). EVTM appeared to serve as a focal mechanism for determining different outcomes such as affective experiences, engagement behavior, and self-perceptions in Korean PA contexts. In particular, most EVTM components identified in this meta-analysis, including expectancy beliefs, attainment value, intrinsic value, utility value, and task values, contributed to engagement behavior, such as persistence, intention to engage, skill practice, and PA. Individuals seemed to be motivated to participate in PA by assessing their perceived ability to succeed and the level of importance, interest, and utility of the task. It is, in fact, well documented in international research that EVTM plays a critical role in engagement behavior. Previous studies (e.g., Chen and Chen, 2012; Shang et al., 2023) substantiated the positive influence of expectancy beliefs and task values on persistence. With the empirical evidence, the results of this study suggest that the positive linkage between expectancy beliefs and task values and engagement behavior may extend beyond Korean PA contexts and apply to various international PA contexts.

Expectancy beliefs, intrinsic value, and task values influenced affective experiences. Individuals who think they were able to perform a given task well and view the task as valuable and enjoyable were likely to promote positive affective experiences, such as satisfaction and interest, in the context. These findings appear to indicate that individuals’ motivational appraisal leads to affective experiences. While EVT has conceptualized affective experiences as memories shaped in the past, which influence motivation (Eccles and Wigfield, 2024), the findings of this study demonstrated the reversed direction. EVTM may instead shape affective experiences, reflecting a unique feature in the Korean PA context. This reversed direction may be understood through control-value theory, a theoretical framework expanded from EVT that emphasizes individuals’ affective experiences or emotions in an achievement context (Pekrun, 2006). This theory assumes that appraisals of ability to succeed or fail (expectancy beliefs) and appraisals of intrinsic and extrinsic values (task values) determine one’s emotions for a given task. From this theoretical perspective, the finding suggests that affective experiences are central to shaping EVTM that may result in positive outcomes in PA contexts. Further exploration is needed to clarify the mediating role of affective experiences in the relationship between ETM and its outcomes.

Only expectancy beliefs served as contributors to self-perceptions. Although this finding is based on a correlation rather than a causal relationship, it is reasoned that individuals with higher expectancy beliefs are likely to foster a strong sense of self, related to their ability, from the theoretical and prior empirical evidence. This finding suggests that self-perceptions are at a more general level than expectancy beliefs (Eccles and Wigfield, 2024). The result may indicate expectancy beliefs as domain-specific, while self-perceptions as task-specific. For example, individuals who believe they successfully perform various skills and PAs may think they have the ability to perform specific tasks given in the context. Connecting the relationship between expectancy beliefs and self-perceptions to the relationship between self-perceptions and task values as discussed in the earlier section, this finding suggests a pathway from domain-specific expectancy beliefs to task-specific values through task-specific self-perceptions. This pathway may reflect a unique pattern of specificity within EVTM in Korean PA contexts, though it should be proven in future research.

The subgroup analysis demonstrated a moderate-to-strong moderating effect of PA contexts in the contributive relationship between EVTM and outcomes. EVTM was consistently related to engagement behavior across all contexts. This consistent pattern suggests EVT is a powerful framework for explaining engagement behavior across different PA contexts beyond South Korea, as evidenced by previous international studies (e.g., Chen and Chen, 2012; Shang et al., 2023). However, the contribution of EVTM to affective experiences was found solely in secondary school PE. This finding seems to reflect a context-specific feature of Korean secondary school PE classes, in which affective experiences are often highlighted to promote PA. According to Lee (2019), in Korean PE classes, students are given opportunities to experience, express, and regulate their emotions in the learning process. In this context, affect-related learning opportunities in relation to EVTM may be functionally and intentionally designed, representing a cultural uniqueness. Caution, however, should be taken when interpreting these results, given the limited sample sizes.

### 4.3 Overall implications

By analyzing the relationships between EVTM and its determinants and outcomes, this meta-analysis provides empirical evidence for EVTM applied to Korean PA contexts. EVTM appears to be influenced by multiple determinants, such as motivational constructs, self-perceptions, social-contextual factors, and task difficulty, contributing to affective experiences, behavioral engagement, and self-perceptions. EVTM may function as an integrated mechanism that links internal and external determinants to motivational consequences in PA contexts. This function of EVTM is consistent with the foundation of EVT and international research findings, suggesting that it is not limited to the Korean context but rather reflects the cross-cultural nature (Eccles and Wigfield, 2024; Shang et al., 2023).

In addition, this study seems to exhibit unique patterns of EVTM in the Korean PA contexts. For example, self-perceptions were influenced by expectancy beliefs, which contributed to task values. Affective experiences functioned as an outcome of EVTM. Although these structural associations have been little addressed in the international literature, they, appear to reflect motivational dynamics of EVTM specific to Korean PA contexts, emphasizing the culturally sensitive or situation-specific nature of motivation (Eccles and Wigfield, 2024). These findings suggest that EVTM operates as flexible depending on the culture rather than merely as cross-transferable. However, it should be mentioned with caution that these findings are not confirmed until cross-cultural comparison studies are carried out, considering that this study included only research conducted within the Korean PA contexts.

Based on the subgroup analyses, the findings can be applied to promote various outcomes in PA contexts. For example, PE teachers and sports coaches in Korea could design challenging but achievable tasks by adjusting the levels of task difficulty, providing step-by-step progression, and giving individualized feedback. These instructional approaches would create a supportive climate, promote individuals’ EVTM and, accordingly, facilitate positive affective experiences as well as PA in secondary school PE and a structured training/exercise context. However, as seen in the results, many of the relationships of EVTM components with relevant determinants and outcomes were not explored in elementary school PE, college PE, and recreational center PA, calling for further research on the function of EVTM.

### 4.4 Limitations and future research

Although this study provides critical insights into determinants and outcomes of EVTM in PA contexts, it also has several limitations. First, the findings may not be interpreted beyond the Korean context because all the data used in the analysis were obtained from studies conducted in Korean PA contexts, even though many of the findings were aligned with patterns observed in international research. Studying cross-cultural comparisons may help clarify cultural influences on EVTM. Second, causal relationships between EVTM and its determinants and outcomes cannot be confirmed due to the correlational nature of the data. However, the directions of the relationships were determined based on those reported in the included studies and inferred from theoretical conceptualizations, if not applicable. This approach provides a theoretically and empirically grounded basis for interpreting the directionality of the associations. Third, there may be potential moderators unexplored in this study. This study used only the type of PA context as a moderator due to insufficient data. As suggested by substantial heterogeneity in effect sizes, there may be other moderators, such as research design, population characteristics (e.g., gender), and measurement instruments. It is necessary to determine different moderators contributing to the relationships between EVTM and its determinants and outcomes. Lastly, the omission of describing sample size justification the included studies not only limits the robustness of the pooled effect estimates but also reflects a potential weakness in the research design. Future studies should clearly describe the process and methods used for sample size justification, including the statistical power of the study to detect true effects reliably.

## 5 Conclusion

This meta-analysis reviewed determinants and outcomes of EVTM in PA contexts in South Korea. The findings demonstrated moderate-to-strong effects of the relationships between EVTM and its determinants and outcomes, suggesting that EVTM was shaped by different internal and external factors, such as motivational constructs, social-contextual influences, and task difficulty, and contributed to affective experiences and behavioral engagement. Self-perceptions were likely to serve as a determinant of expectancy beliefs and an outcome of task values. Overall, these findings suggest both cross-cultural patterns and distinct mechanisms of EVTM in PA contexts. Future research should address cross-cultural comparisons to confirm the influence of cultural differences on the mechanism of EVTM.

## Data Availability

The original contributions presented in the study are included in the article/Supplementary material, further inquiries can be directed to the corresponding author.
