# Supplementary material for: Expectancy-value theories applied in Korean physical activity contexts: a meta-analysis
Source: Front Psychol. 2025 Sep 16;16:1678503. doi: 10.3389/fpsyg.2025.1678503 (PMC12479517; doi:10.3389/fpsyg.2025.1678503)
Supplement: Supplementary file 1 [file Table_1.docx]

Supplementary Table Characteristics of the included studies

| Year | Author | Context | #Sample | Gender | Analysis | Determinant | Outcome |
| --- | --- | --- | --- | --- | --- | --- | --- |
| Studies on determinant–EVTM pairs | | | | | | | |
| 2023 | Kim & Yoon | Structured training/exercise | 262 | Mixed | Regression | Self-perceptions | Expectancy beliefs, cost |
| 2023 | Son & Yang | College PE | 265 | Mixed | Regression | Social-contextual factors | Expectancy beliefs, attainment value, intrinsic value, utility value |
| 2020 | Baek et al. | Seceondary PE | 400 | Mixed | Regression | Self-perceptions | Expectancy beliefs, task values |
| 2022 | Kwak et al. | Structured training/exercise | 203 | Mixed | SEM | Self-perceptions | Expectancy beliefs, task values |
| 2020 | Jung & Lee | Elementary PE | 507 | Mixed | SEM | Motivational constructs, social-contextual factors | Attainment value, intrinsic value, utility value |
| 2020 | Kim | Elementary PE | 467 | Mixed | SEM | Motivational constructs, social-contextual factors | Attainment value, intrinsic value |
| 2019 | Jung | Elementary PE | 507 | Mixed | SEM | Social-contextual factors | Utility value |
| 2017 | Kang | Structured training/exercise | 320 | Mixed | SEM | Self-perceptions | Attainment value, intrinsic value, utility value |
| 2017 | Kang et al. | Structured training/exercise | 338 | Reported by gender | SEM, LCA | Self-perceptions | Expectancy beliefs, task values |
| 2016 | Kim | Seceondary PE | 379 | Mixed | SEM | Self-perceptions | Expectancy beliefs, task values |
| 2014 | Do & Yoo | Seceondary PE | 336 | Mixed | SEM | Motivational constructs | Expectancy beliefs, aattainment value, intrinsic value, utility value |
| 2013 | Choi | Elementary PE | 664 | Female | SEM | Self-perceptions | Attainment value, intrinsic value, utility value |
| 2012 | Park & Kim | Structured training/exercise | 454 | Male | SEM, MGSEM | Self-perceptions | Utility value, cost |
| 2012 | Park & Yoo | Seceondary PE | 778 | Mixed | SEM | Motivational constructs | Expectancy beliefs |
| 2012 | Park | Seceondary PE | 760 | Mixed | SEM, MGSEM | Motivational constructs, task difficulty | Task values |
| 2011a | Park & Lee | Seceondary PE | 726 | Mixed | Regression | Motivational constructs, task difficulty | Expectancy beliefs, task values |
| 2011b | Park & Lee | Seceondary PE | 255 | Mixed | SEM | Motivational constructs | Expectancy beliefs, task values |
| 2010 | Park | Seceondary PE | 524 | Mixed | SEM | Motivational constructs | Task values |
| Studies on EVTM–outcome pairs | | | | | | | |
| 2024 | Son & Yang | Recreational Center PA | 155 | Mixed | Regression | Expectancy beliefs, attainment value, intrinsic value, utility value | Engagement behavior |
| 2024 | Yun | College PE | 254 | Mixed | SEM | Expectancy beliefs, task values | Engagement behavior, affective experiences |
| 2023 | Kim & Yoon | Structured training/exercise | 262 | Mixed | Regression | Expectancy beliefs, task values, cost | Engagement behavior, self-perceptions |
| 2023 | Lee | College PE | 368 | Mixed | SEM | Expectancy beliefs, task values, cost | Engagement behavior, self-perceptions |
| 2023 | Seo | College PE | 346 | Mixed | Regression | Expectancy beliefs, intrinsic value, utility value | Engagement behavior, self-perceptions |
| 2020 | Baek et al. | Seceondary PE | 400 | Mixed | Regression | Expectancy beliefs, task values | Affective experiences |
| 2020 | Jung & Lee | Elementary PE | 507 | Mixed | SEM | Attainment value, intrinsic value, utility value | Engagement behavior |
| 2020 | Kim | Elementary PE | 467 | Mixed | SEM | Attainment value, intrinsic value | Engagement behavior |
| 2019 | Jung | Elementary PE | 507 | Mixed | SEM | Attainment value, intrinsic value, utility value | Engagement behavior |
| 2019 | Lee | Seceondary PE | 901 | Mixed | SEM | Expectancy beliefs, task values | Affective experiences |
| 2019 | Song | Seceondary PE | 353 | Mixed | SEM | Expectancy beliefs, task values | Engagement behavior, self-perceptions |
| 2019 | Yang | College PE | 318 | Female | Regression | Expectancy beliefs, aattainment value, intrinsic value, utility value | Engagement behavior |
| 2018 | Choi et al. | College PE | 814 | Mixed | Regression | Expectancy beliefs, Attainment value, intrinsic value, task values | Engagement behavior |
| 2017 | Kang | Structured training/exercise | 320 | Mixed | SEM | Attainment value, intrinsic value, utility value | Engagement behavior |
| 2017 | Kang et al. | Structured training/exercise | 338 | Reported by gender | SEM, LCA | Expectancy beliefs, task values | Engagement behavior |
| 2016 | Jung | Elementary PE | 439 | Mixed | SEM | Expectancy beliefs, attainment value, intrinsic value, utility value | Engagement behavior |
| 2016 | Kim | Seceondary PE | 379 | Mixed | SEM | Expectancy beliefs, task values | Motivational constructs |
| 2015 | Cho | Seceondary PE | 371 | Mixed | Regression | Expectancy beliefs, task values | Affective experiences, motivational constructs |
| 2015 | Jung & Nam | Seceondary PE | 783 | Mixed | Regression | Expectancy beliefs, attainment value, intrinsic value, utility value | Engagement behavior, affective experiences |
| 2014 | Do & Yoo | Seceondary PE | 336 | Mixed | SEM | Expectancy beliefs, attainment value, intrinsic value, utility value | Engagement behavior |
| 2013 | Song & Heo | Seceondary PE | 707 | Mixed | SEM, MGSEM | Expectancy beliefs, task values | Motivational constructs |
| 2012 | Park & Kim | Structured training/exercise | 454 | Male | SEM, MGSEM | Expectancy beliefs, attainment value, intrinsic value, utility value, cost | Engagement behavior |
| 2012 | Park & Yoo | Seceondary PE | 778 | Mixed | SEM | Expectancy beliefs | Engagement behavior, affective experiences, self-perceptions |
| 2012 | Park | Seceondary PE | 760 | Mixed | SEM, MGSEM | Task values | Engagement behavior, learning achievement |
| 2011a | Park & Lee | Seceondary PE | 726 | Mixed | Regression | Expectancy beliefs, task values | Engagement behavior |
| 2011b | Park & Lee | Seceondary PE | 255 | Mixed | SEM | Expectancy beliefs, task values | Engagement behavior, affective experiences |
| 2010 | Park | Seceondary PE | 524 | Mixed | SEM | Task values | Engagement behavior, motivational constructs, affective experiences |

LCA, latent class analysis; MGSEM, multi-group structural equation modeling; PA, physical activity; PE, physical education; SEM, structural equation modeling.
